# Frequency-tuned electromagnetic field therapy improves post-stroke motor function: A pilot randomized controlled trial

**DOI:** 10.3389/fneur.2022.1004677

**Published:** 2022-11-14

**Authors:** Batsheva Weisinger, Dharam P. Pandey, Jeffrey L. Saver, Arielle Hochberg, Adina Bitton, Glen M. Doniger, Assaf Lifshitz, Ofir Vardi, Esther Shohami, Yaron Segal, Shira Reznik Balter, Yael Djemal Kay, Ariela Alter, Atul Prasad, Natan M. Bornstein

**Affiliations:** ^1^BrainQ Technologies, Ltd., Jerusalem, Israel; ^2^Manipal Hospital Physiotherapy and Rehabilitation, New Delhi, India; ^3^Department of Neurology, UCLA Comprehensive Stroke and Vascular Neurology Program, David Geffen School of Medicine, University of California, Los Angeles, Los Angeles, CA, United States; ^4^Hebrew University of Jerusalem, Jerusalem, Israel; ^5^Department of Neurology, B. L. Kapur Super Specialty Hospital (BLK), National Capital Territory of Delhi, New Delhi, India; ^6^Brain Division, Shaare Zedek Medical Center, Jerusalem, Israel

**Keywords:** ischemic stroke, ELF-EMF, ENTF, neurostimulation, NIBS, magnetic field therapy, upper extremity motor function, neurorecovery

## Abstract

**Background and purpose:**

Impaired upper extremity (UE) motor function is a common disability after ischemic stroke. Exposure to extremely low frequency and low intensity electromagnetic fields (ELF-EMF) in a frequency-specific manner (Electromagnetic Network Targeting Field therapy; ENTF therapy) is a non-invasive method available to a wide range of patients that may enhance neuroplasticity, potentially facilitating motor recovery. This study seeks to quantify the benefit of the ENTF therapy on UE motor function in a subacute ischemic stroke population.

**Methods:**

In a randomized, sham-controlled, double-blind trial, ischemic stroke patients in the subacute phase with moderately to severely impaired UE function were randomly allocated to active or sham treatment with a novel, non-invasive, brain computer interface-based, extremely low frequency and low intensity ENTF therapy (1–100 Hz, < 1 G). Participants received 40 min of active ENTF or sham treatment 5 days/week for 8 weeks; ~three out of the five treatments were accompanied by 10 min of concurrent physical/occupational therapy. Primary efficacy outcome was improvement on the Fugl-Meyer Assessment – Upper Extremity (FMA-UE) from baseline to end of treatment (8 weeks).

**Results:**

In the per protocol set (13 ENTF and 8 sham participants), mean age was 54.7 years (±15.0), 19% were female, baseline FMA-UE score was 23.7 (±11.0), and median time from stroke onset to first stimulation was 11 days (interquartile range (IQR) 8–15). Greater improvement on the FMA-UE from baseline to week 4 was seen with ENTF compared to sham stimulation, 23.2 ± 14.1 vs. 9.6 ± 9.0, *p* = 0.007; baseline to week 8 improvement was 31.5 ± 10.7 vs. 23.1 ± 14.1. Similar favorable effects at week 8 were observed for other UE and global disability assessments, including the Action Research Arm Test (Pinch, 13.4 ± 5.6 vs. 5.3 ± 6.5, *p* = 0.008), Box and Blocks Test (affected hand, 22.5 ± 12.4 vs. 8.5 ± 8.6, *p* < 0.0001), and modified Rankin Scale (−2.5 ± 0.7 vs. −1.3 ± 0.7, *p* = 0.0005). No treatment-related adverse events were reported.

**Conclusions:**

ENTF stimulation in subacute ischemic stroke patients was associated with improved UE motor function and reduced overall disability, and results support its safe use in the indicated population. These results should be confirmed in larger multicenter studies.

**Clinical trial registration:**

https://clinicaltrials.gov/ct2/show/NCT04039178, identifier: NCT04039178.

## Introduction

Stroke is a leading cause of adult disability worldwide (1–3). While early reperfusion interventions improve outcomes (4), they are delivered to a small proportion of patients, leaving many stroke survivors with residual disabilities, impairments and dependency on others. This is accompanied by a large economic burden on both a personal and societal level, as a result of direct medical cost, as well as indirect costs due to underemployment and premature death (5).

Beyond the acute phase, standard of care focuses on rehabilitation through a coordinated effort of medical, social, educational, and vocational approaches to retrain an individual with newly acquired disabilities (6). Effective rehabilitation programs employ highly intensive and repetitive physical therapy to enhance neurologic recovery (7), possibly via direct influence on functional reorganization in the brain (i.e., plasticity). However, there is considerable variability among facilities in the implementation of therapeutic approaches that maximize functional recovery (8–11). Further, despite receiving standard rehabilitation care, many patients are left with lifelong disabilities and impairments, never returning to their pre-stroke ability level. For such people, one of the most common and persistent disabling symptoms is hemiparesis and upper limb motor impairment (12–14).

In the subacute post-stroke phase, and in response to both the initial, primary injury and the ensuing secondary injury cascade, the central nervous system (CNS) attempts to repair and reorganize itself via the secretion of survival-promoting agents (such as growth factors and anti-inflammatory cytokines), recovering damaged networks, and sprouting collateral synaptic connections to restore motor and cognitive functions (15–18). In preclinical models, neuroplasticity can be altered by external factors, including pharmacologic agents, electrical stimulation, and environmental stimulation (18).

Non-invasive brain stimulation (NIBS) techniques have demonstrated the capacity to enhance neuroplasticity in preclinical models, and have shown evidence suggestive of recovery in clinical trials (19–21). NIBS methods include repetitive transcranial magnetic stimulation (rTMS; (22)), transcranial direct current stimulation (tDCS; (23)), vagal nerve stimulation (24), and extremely low frequency, low intensity electromagnetic fields (ELF-EMF). While many such methods have been used with relative success, the limited applicability and strict usability requirements are such that none have yet to qualify as a standard of care treatment. ELF-EMF is a promising noninvasive therapeutic technique for post-stroke care, shown in preclinical studies to exert a beneficial effect on many of the cellular processes that modulate damage and recovery post-stroke, including calcium signaling, oxidative stress, and inflammatory response (25, 26). In a randomized clinical trial, ELF-EMF in the subacute post-stroke period was associated with increased enzymatic antioxidant activity, reduced oxidative stress, and improved performance on standardized assessments of activities of daily living, cognition, and mood (27, 28). Accordingly, and due to its relative safety and wide range of applicability, ELF-EMF treatment in the subacute phase may be a viable post-stroke therapy.

A novel ELF-EMF technique is a non-invasive, brain computer interface-based (BCI-based), low frequency, low intensity, frequency-tuned EMF therapy (Electromagnetic Network Targeting Field therapy; ENTF therapy), designed to expose impaired neuronal networks to oscillating fields similar to those of the CNS, in an effort to promote network reorganization post-injury. The human brain is organized into complex functional networks (29); healthy activity in the CNS results from the synchronization of thousands of individual neurons in the form of sophisticated and organized oscillations. These synchronous global oscillations within specific frequency bands represent functionally connected neural networks, which generate electrical activity measurable with electrophysiological techniques, and are a fundamental part of the functionality of the brain (30, 31). These oscillatory patterns are correlated with cognitive states, motor functions, and electrophysiological activity both within and beyond the CNS. Changes in these patterns have been observed following ischemic stroke, and after other nervous system disorders like traumatic brain injury (31–33).

Neural network dynamics are sensitive to endogenous (34, 35) and exogenous electric and magnetic fields at specific frequencies (35–37), and oscillating ELF-EMF fields are hypothesized to promote the return of synchronization and network reorganization within the targeted networks. The motivation behind the present study (BQ3) is the possibility that ELF-EMF exposures can specifically target these impaired networks by exposing such networks to oscillating fields similar to those that characterize a healthy CNS, in order to promote network reorganization post-injury. The treatment protocol is based upon the most prominent frequencies of these motor-related oscillations, extracted using advanced machine learning algorithms from electrophysiological recordings of large populations of healthy and impaired individuals performing motor tasks (35).

In a preclinical rodent stroke model, oscillating ELF-EMF stimulation was associated with decreased edema, increased white matter integrity, and evidence of neural regeneration (38). Additionally, initial data from ongoing preclinical collaborative studies (unpublished) using this technique indicate changes in measures of oxidative stress, inflammation, and cell death. Overall, data suggest that such treatment targets cellular pathways comprising functional neural networks, promotes neural plasticity, and modulates the secondary injury cascade, all of which aid clinical recovery.

Accordingly, a pilot randomized, double-blind, sham-controlled trial of a BCI-based, low frequency, low intensity, frequency-tuned ENTF therapy to improve upper extremity motor function and reduce disability in subacute post-stroke patients was designed and executed. Greater improvement in upper extremity motor function is expected in individuals who receive ENTF treatment, as compared to those in the sham control group.

## Materials and methods

### Study design and participants

This study, the BQ3 trial (NCT04039178), was a prospective, randomized, double-blind, sham-controlled study. See Supplementary Table S1 for full study entry criteria.

The trial was conducted at the BLK Super Specialty Hospital, New Delhi, India, a multi-specialty private hospital accredited by the Joint Commission International. Study operations were overseen by JSS Medical Research, an international, full-service contract research organization. The hospital institutional review board provided ethics approval, and written informed consent was obtained from all participants.

The main inclusion criteria for this study were: patients 4–21 days post-ischemic stroke with first stroke or no prior upper extremity impairment, right hand dominant, with a Fugl-Meyer Assessment – Upper Extremity (FMA-UE) score between 10 and 45. Patients were also screened for their ability to participate in the treatment procedures based on their ability to be seated for 70 consecutive minutes, and follow a three-step command. Patients who were not medically stable, with a physiological, neurological, or psychiatric history that might confound study measures, or contraindications for MRI scanning were not considered for this study.

### Randomization and blinding

The study was planned for 50 participants, the first four of whom would be assigned directly to the treatment group (run-in phase). The remaining 46 participants were to be randomly assigned to active ENTF or sham stimulation (randomized phase) in a 1:1 ratio (block randomization; SAS-generated), by an individual not otherwise associated with the study. After determining group allocation, the individual keyed in group assignment to the device (required only once per participant). Participants and study staff were not aware of group assignment. The device does not produce any perceptible light, sound, or sensation during the ELF-EMF activity; sham stimulation consists of the same general treatment flow, but with the wave generator inactive during the session, and as governed by the group assignment saved for each participant within the device. Thus, experience with the BQ device during sessions was the same irrespective of group assignment, allowing for proper blinding.

### Materials

Treatment was administered with a proprietary BCI-based stimulation device (BQ 1.0; BrainQ Technologies Ltd., Jerusalem, Israel; product manual available upon request), exposing the entire brain and the cervical and upper thoracic portion of the spinal cord to the ENTF. The device technology uses machine learning algorithms (Python, 3.6) to identify high resolution spectral patterns that characterize motor functions within EEG measurements recorded during functional motor tasks. For this custom-made algorithm, EEG data from healthy and unhealthy individuals was collected while executing discreet motor tasks. A novel normalization technique was used to reduce inter-subject variability. Machine learning models were used to differentiate between healthy and unhealthy data traces. The explanatory features used by these models to generate their decisions were then used to inform a non-invasive and frequency-specific, extremely low frequency (1–100 Hz), low intensity (< 1 Gauss) electromagnetic field treatment applied to a participant's CNS, delivered via a magnetic coil. The device emitted ELF-EMF only for participants in the ENTF group but not for those in the sham group.

### Procedure

ENTF or sham therapy was provided 5 days a week for ~8 weeks, for a total of 40 treatment sessions. During each treatment session, participants received 40 minutes of treatment with the BQ device (active or sham). During ~3 weekly sessions, concurrent with the ENTF or sham therapy, participants performed 10 minutes of upper extremity physical therapy/occupational therapy-based exercises (e.g., gripping a ball, reaching) with the guidance of a therapist. Separate from the treatment sessions, participants also received ~1 h of physical therapy per day throughout their participation as part of the hospital's standard clinical regimen.

### Outcome measures

The primary clinical efficacy outcome was change in upper extremity motor function from baseline to end of treatment (week 8), measured with the FMA-UE, a performance-based impairment index designed to assess motor function, balance, sensation and joint function in patients with post-stroke hemiparesis (39, 40). FMA-UE was assessed throughout the course of treatment at baseline, week 4, week 8 and week 12. However, due to early trial closure because of the COVID-19 pandemic, follow-up assessments at week 12 were not completed for many participants (available data for < 80%), so analyses included only changes at week 4 and week 8.

Secondary clinical efficacy outcomes were: Action Research Arm Test [ARAT, coordination, dexterity, and function (41)]; Box & Blocks Test [BBT, gross manual dexterity (42)]; Fugl-Meyer Assessment – Lower Extremity (FMA-LE) (39, 40); modified Rankin Scale (mRS) of global disability (43); National Institutes of Health Stroke Scale [NIHSS, stroke-related neurological deficit (44)]; Patient-Reported Outcome Measurement Information System Global 10 [PROMIS-10, patient-reported assessment of global health and quality of life (45)]. Notably, some prespecified outcome measures were not analyzed due to < 80% valid data. These include: cognitive measures [Trail Making Test (46); Montreal Cognitive Assessment (47)], as they were administered in English, which was not most participants' primary language; imaging (MRI), because of variability in scan parameters due to use of multiple scanners; and blood biomarkers, as some growth factors were out of the detection range for many subjects (28, 48). EEG was collected as an additional, exploratory endpoint, and was analyzed separately from the clinical results; these results are reported elsewhere (49).

The primary safety outcome was adverse events during the trial period.

### Statistical analyses

Statistical analyses were conducted with SAS V9.4 (SAS Institute, Cary NC, USA). For behavioral outcomes, continuous variables were generally summarized by mean and standard deviation (SD), and categorical variables by percentage. Changes from baseline in continuous outcomes were analyzed by analysis of variance (ANOVA) or repeated measures ANOVA (SAS PROC MIXED), with each outcome modeled as a function of treatment group; if >1 post-baseline visit, each outcome was also modeled as a function of visit, as well as the treatment group^*^visit interaction. Baseline value for the respective outcome was entered as a covariate to adjust for variation in baseline score between the groups. LSmeans (model estimated means) per group and the differences between the groups were estimated from the models, along with respective levels of significance. If >1 post-baseline visit, then the treatment group^*^visit interaction term was the parameter of interest, and the LSmeans per group, as well as the differences between the groups at each visit, were estimated from the models, along with respective levels of significance. Continuous demographic and baseline data were compared between the groups with a Wilcoxon two-sample test. Categorical variables were compared using Fisher's exact test when the response was binary, and with a Cochran Armitage trend test when the response was ranked (e.g., mRS). Nominal two-sided *p* values are presented without *post-hoc* testing to correct for multiple comparisons given that this was a pilot study.

As a pilot study, sample size justification was formulated based on the literature regarding the study's primary outcome, as well as the treatment characteristics of the target population (50). The sample size calculation assumed 80% power, a 5-point difference between groups on the FMA-UE, and a SD of 6, resulting in a study with 46 participants. The study was designed to include 46 randomized participants, preceded by 4 treatment-only run-in participants, for a total sample size of 50.

The prespecified primary analysis set for efficacy evaluation was the per protocol (PP) set, defined as all participants who were randomized and participated in 80% of the treatment visits, were not absent for more than 5 consecutive visits, and completed the week 8 outcome evaluation; the PP set consisted of 8 participants allocated to sham stimulation, and 13 to ENTF stimulation. Participant characteristics were evaluated in the intention to treat (ITT) analysis set, defined as all randomized participants who completed at least one treatment session (sham or active); the ITT set consisted of 9 sham and 15 ENTF participants. The as treated (AT) safety set, defined as all participants (including the *n*=4 treatment-only run-ins) who received at least one treatment session, consisted of 9 sham and 19 ENTF participants.

## Results

**Figure 1:**
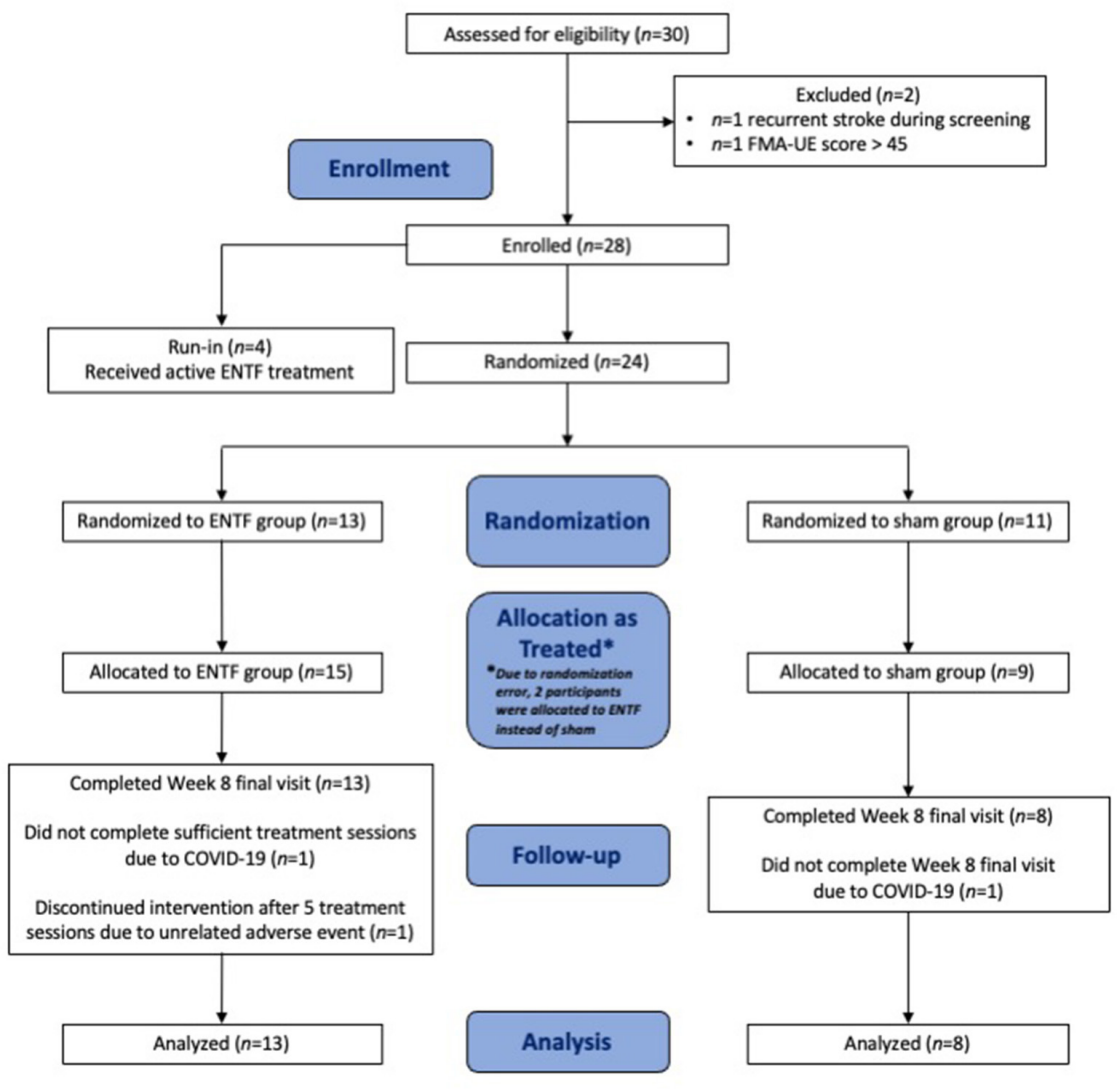
Participant study flow diagram.

The study was conducted between first enrollment on December 11, 2018 and final study visit on March 21, 2020. As emergence of the COVID-19 pandemic precluded continuation of study operations, the study was discontinued early, and data was analyzed after enrollment of 4 run-in and 24 randomized participants. Participant study flow diagram is shown in Figure 1.

Participant baseline characteristics in the PP set are shown in Table 1. Participant characteristics were generally well balanced across the treatment groups. Participant baseline characteristics in the ITT and the AT safety sets were similar to the PP set (Supplementary Tables S2, S3).

**Table 1:** Participant demographics^*^.

	**Sham Group (*n* = 8)**	**ENTF Group (*n* = 13)**	**Total (*n* = 21)**
Age, yrs, mean (±SD)	55.3 (±10.1)	54.3 (±17.8)	54.7 (±15.0)
Sex, female (%)	25%	15%	19%
Race-Ethnicity, South-Asian (%)	100%	100%	100%
Hand dominance, right (%)	100%	100%	100%
Affected hand, right (%)	63%	38%	48%
Time from stroke onset to first treatment, days, median (IQR)	14.0 (10.8–16.0)	9.0 (7.0–14.0)	11.0 (8.0–15.0)
FMA-UE Baseline, mean (±SD)	18.8 (±8.7)	26.8 (±11.5)	23.7 (±11.0)
mRS Baseline, mean (±SD)	3.4 (±0.7)	3.6 (±0.5)	3.5 (±0.6)

* No significant differences were noted between groups at baseline.

### Efficacy outcomes

**Figure 2:**
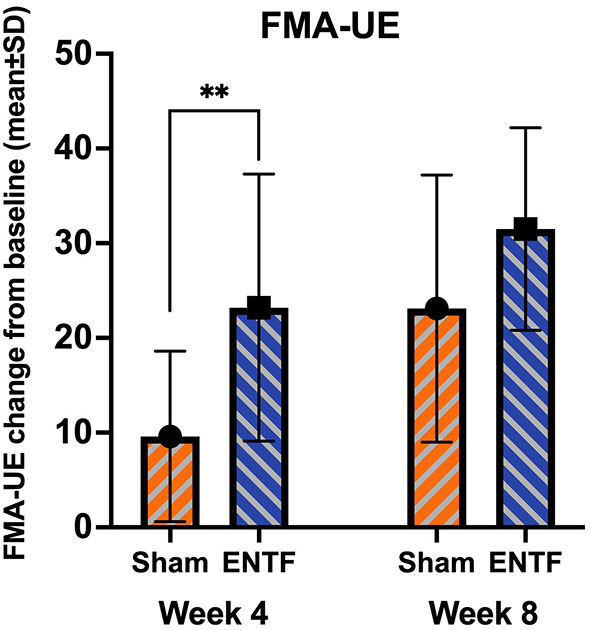
Fugl-Meyer Assessment – Upper Extremity (FMA-UE) score changes from baseline to week 4 and week 8 (range: 0-66; 66 is greatest mobility/optimal recovery). FMA-UE absolute score change from baseline to week 4 (sample mean, error bars correspond to SD; significance based on difference in LSmeans) was significantly greater for the ENTF group than sham group (23.2 ± 14.1 vs. 9.6 ± 9.0; *p* = 0.007; ** < 0.01). Absolute score change from baseline to week 8 was also greater, though not significantly so, for the ENTF group than sham group (31.5 ± 10.7 vs. 23.1 ± 14.1; *p* = 0.06).

For FMA-UE (primary clinical efficacy outcome measure), performance score improvements were greater in the ENTF compared to the sham group throughout treatment, both at week 4 (*p* = 0.007) and week 8 (Table 2; Figure 2). In terms of absolute numerical values, a ceiling effect was noted at week 8. In the active stimulation group, 77% of participants attained scores in the top 10% of the scale, including 46% receiving the highest score (66/66). By comparison, only 38% of the sham group attained scores in the top 10% and no participants attained the highest score.

**Table 2:** Efficacy outcome measures^*^.

	**Sham group (*n* = 8)**	**ENTF group (*n* = 13)**	Significance^**^
Primary outcome measure			
FMA-UE Week 4	9.6 ± 9.0	23.2 ± 14.1	0.007
FMA-UE Week 8	23.1 ± 14.1	31.5 ± 10.7	0.06
Secondary outcome measures			
mRS Week 8	−1.3 ± 0.5	−2.5 ± 0.7	0.0005
ARAT Grasp, Week 8	8.1 ± 7.6	9.1 ± 6.8	0.15
ARAT Grip, Week 8	5.4 ± 4.1	6.8 ± 3.7	0.13
ARAT Pinch, Week 8	5.3 ± 6.5	13.4 ± 5.6	0.008
ARAT gross movement, Week 8	3.8 ± 2.1	2.8 ± 1.9	0.50
ARAT total score, Week 8	22.5 ± 17.1	32.1 ± 14.2	0.09
BBT (Affected hand), Week 2	0.5 ± 0.8	1.3 ± 1.7	0.07
BBT (Affected hand), Week 4	1.4 ± 1.5	6.8 ± 5.5	0.08
BBT (Affected hand), Week 6	3.3 ± 3.6	10.9 ± 7.1	0.02
BBT (Affected hand), Week 8	8.5 ± 8.6	22.5 ± 12.4	< 0.0001
BBT (Non-affected hand), Week 2	2.9 ± 0.6	1.8 ± 2.0	0.04
BBT (Non-affected hand), Week 4	4.4 ± 2.1	9.0 ± 5.0	0.08
BBT (Non-affected hand), Week 6	7.8 ± 2.1	12.4 ± 5.4	0.08
BBT (Non-affected hand), Week 8	9.4 ± 3.9	18.4 ± 7.4	0.0003
FMA-LE, Week 8	9.9 ± 6.5	13.8 ± 7.8	0.03
NIHSS, Week 8	−4.8 ± 3.2	−6.6 ± 3.4	0.03
PROMIS-10 global physical health, Week 8	9.0 ± 5.2	11.0 ± 6.8	0.33
PROMIS-10 global mental health, Week 8	13.0 ± 7.2	14.7 ± 6.1	0.48

* All values represent change from baseline (mean±SD).

** From LSmean adjusted means.

**Figure 3:**
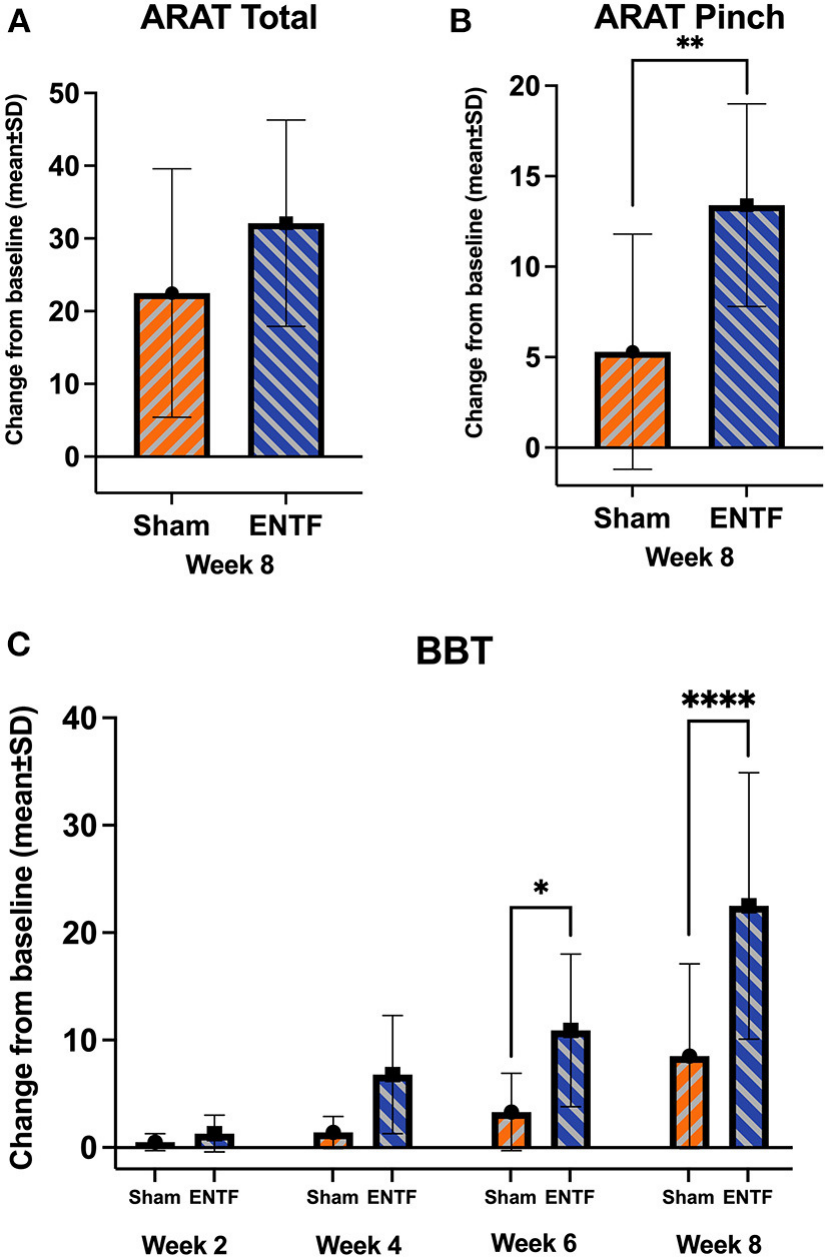
Evolution of upper extremity function secondary efficacy outcomes (sample mean; error bars correspond to SD; significance based on difference in LSmeans). **(A)** Action Research Arm Test (ARAT) total score from baseline to week 8 (absolute score change: ENTF group 32.1 ± 14.2 vs. sham group 22.5 ± 17.1, *p* = 0.09). **(B)** ARAT Pinch subscale score from baseline to week 8 (absolute score change: ENTF 13.4 ± 5.6 vs. sham 5.3 ± 6.5, *p* = 0.008; ** p < 0.01). **(C)** Box and Blocks Test (BBT) affected hand scores from baseline through weeks 2, 4, 6 (absolute change score: ENTF 10.9 ± 7.1 vs. sham 3.3 ± 3.6, *p* = 0.02; *p < 0.05) and 8 (absolute change score: 22.5 ± 12.4 vs. 8.5 ± 8.6, *p* < 0.0001; ****p < 0.0001).

Score changes for secondary efficacy outcomes related to motor function generally reflect greater improvement for ENTF compared with sham control, with limited exception (Table 2). Specifically, greater improvement for ENTF was found for the ARAT total score (Figure 3A) and three out of four of the ARAT subscales, most notably, the Pinch subscale (*p* = 0.008, Figure 3B). Significantly greater improvement was also seen on the BBT in the affected hand (week 6: *p* = 0.02; week 8: *p* < 0.0001, Figure 3C); the non-affected hand also showed greater improvement in the ENTF group. Significantly greater improvement was also seen in the FMA-LE scores changes (Table 2).

**Figure 4:**
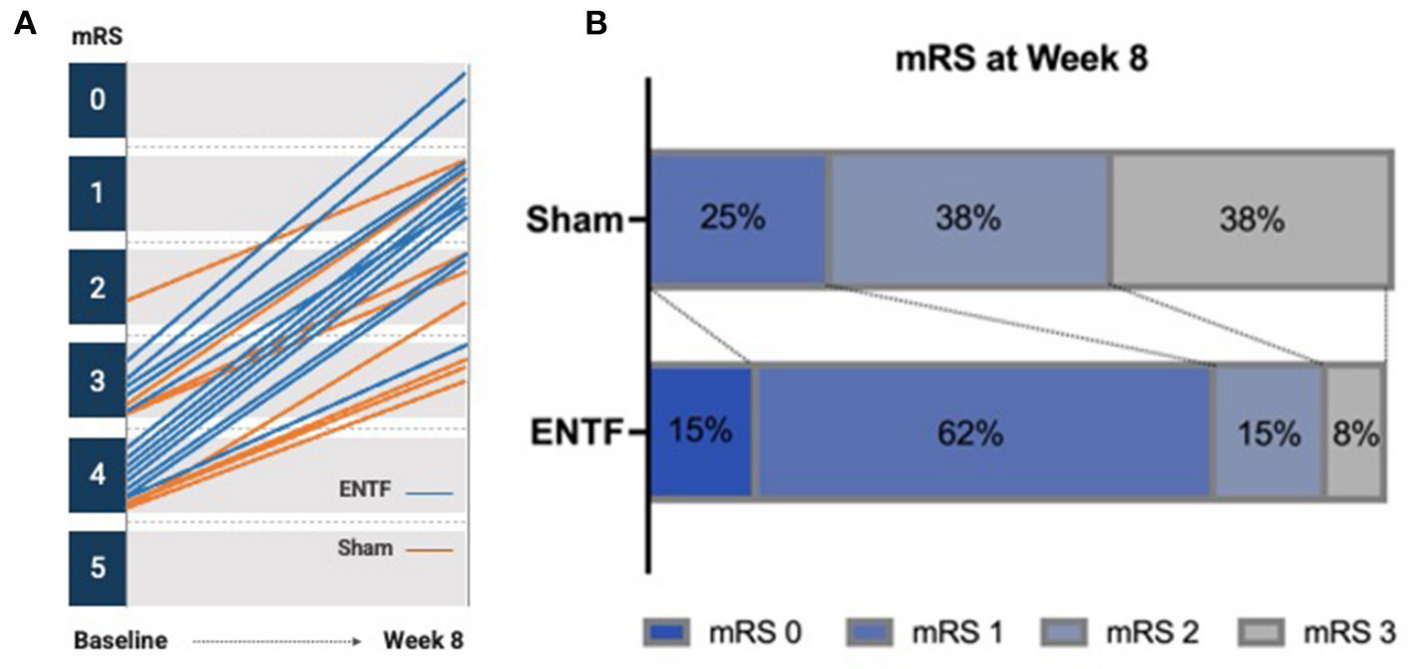
Change in modified Rankin Scale (mRS) from baseline to week 8. **(A)** Individual participant trajectories from baseline to week 8, yielding greater group reduction in disability severity in the ENTF compared to sham group, −2.5 ± 0.7 vs. −1.3 ± 0.5, *p* = 0.0005. **(B)** Distribution of final mRS scores at week 8, with more favorable outcomes evident in ENTF vs. sham group.

In addition, both mRS and NIHSS scores showed greater improvement for the ENTF group. On the mRS, the ENTF group showed significantly greater reduction in degree of disability between baseline and week 8; −2.5 ± 0.7 vs. −1.3 ± 0.5, *p* = 0.0005. Notably, 92% of participants in the ENTF group improved by at least two points compared to only 25% in the sham group (Figure 4A). By week 8, 77% of participants in the ENTF group vs. 25% in the sham group had an mRS score of 1 or 0, indicative of little to no residual disability (Figure 4B). In contrast, patient-reported efficacy outcomes related to generic health-related quality of life (PROMIS-10) did not show a difference in degree of improvement between treatment groups. A descriptive table of raw scores for each of the analyzed clinical outcome measures at all assessment time points is included in Supplementary Table S4.

### Safety outcomes

Two adverse events (AEs) were reported, neither related to the ENTF treatment. There were no device-related infections or unexpected device-related adverse events. Additionally, there were no complaints of discomfort during ENTF treatment.

## Discussion

In this double-blind, randomized, sham-controlled trial, frequency-tuned ELF-EMF stimulation with the BQ device, or ENTF therapy, was associated with enhanced recovery of upper extremity function when initiated in the subacute period and continued for 2 months as compared to sham control. Beneficial results were evident not only for the primary outcome measure (FMA-UE), but also for several other measures of upper extremity function, including the BBT (manual dexterity) and the ARAT (coordination, dexterity, and function). Moreover, ELF-EMF treatment was associated with a greater reduction of global disability in daily activities (mRS). In addition, there was no evidence of safety concerns, and there were no participant complaints of discomfort during treatment.

There was an indication of beneficial effect on lower extremity function in addition to upper extremity function, suggesting a general enhancement of motor function beyond the upper extremity. In contrast, ENTF stimulation was not associated with benefit on a generic measure of mental and physical health-related quality of life. Indeed as the intervention in this study was specifically designed to target motor impairment, it cannot be assumed that generic physical and mental health measures would show differential benefits between the two groups. Still, it should be noted that these were subjective patient-reported ratings.

The magnitude of benefit of ENTF treatment was robust and clinically meaningful across multiple metrics of upper extremity motor function, and especially the FMA-UE (51, 52). Further, the reduction of global disability as assessed by the mRS supports a strong positive effect on overall functioning. The substantial difference in outcomes between active and sham-treated groups was not related to unusually poor performance in the control arm. The degree of improvement on the FMA-UE in the control group was typical of those in prior natural history studies (51, 52). Similarly, in terms of overall stroke disability, the degree of improvement on the mRS in the control group was similar to that in control groups in prior large trials and observational studies (53–57).

EEG recordings (exploratory) of study participants reflected a pattern of brain activity indicative of recovery exclusively in the ENTF group (49). More specifically, the EEG results are consistent with improvement in movement inhibition or motor learning (58) as well as increased signal complexity, a characteristic of healthy brain activity (59). In effect, the EEG data provide evidence for a biomarker of recovery putatively linked to plasticity (59) in the ENTF group but not in the sham group.

At the neuronal level, given the continued degradation of neurons in the days and weeks following a stroke, as well as the secondary injury cascade whereby cells adjacent to the site of injury continue to degrade (60), a non-invasive treatment that targets affected cells and networks during this critical time period and prevents further degradation has great clinical utility, addressing a gap in subacute care options. The most challenging question regarding the effect of ELF-EMF on (neuronal) tissue is in identifying the transduction mechanism by which the applied field and the biological medium interact, achieving such effects. Although the exact mechanism remains unknown, two different steps have to be taken into account when contemplating the mechanism of action of ELF-EMF: 1) the initial interaction between the external magnetic field (MF) and the biological system and 2) the cascade of biological events leading to the physiological/ behavioral effect seen in this as well as other studies, both human and animal (61, 62).

Regarding the initial interaction step, there are two plausible transduction mechanisms: electric currents inducing minor changes in the conductive tissues (unlikely for intensities < 1 G such as used in this study), and possible direct action of the MF on endogenous magnetoreception (63). As for the cascade of biological events that follow, it has been shown that ELF-EMF effects are likely to involve a number of cellular targets such as changes in intracellular Ca^2+^ signaling (64–69), elements of the oxidative stress cascade (70, 71), nitric oxide (72, 73), G-protein receptor coupling (74, 75), and the inflammatory response (76, 77), to name a few. Some of these cellular targets have been identified and described in the studies conducted previously by members of this group, as well as by others in the field, revealing a candidate for the cascade of events that may ultimately give rise to the observed effects on the cellular, network and behavioral levels.

For example, in human neuroblastoma and rat pituitary cells, ELF-EMF exposure increases proliferation and inhibits programmed cell death by up-regulating the expression of voltage-gated Ca^2+^ (Ca_v_) channels [5–1,000 μT and frequencies of 1–100 Hz, (65)]. Additionally, it has been shown that ELF-EMF increases generation and metabolism of nitric oxide (NO) in poststroke patients, promoting cellular processes that support neuroplasticity, and thus may enhance post-stroke rehabilitation (27). Furthermore, ELF-EMF exposure (50 Hz, 1 mT, 1 to 7 h/day for 7 days) significantly enhanced neurogenesis in the dentate gyrus (DG) of adult mice, as demonstrated by increased numbers of cells double-labeled for BrdU and doublecortin (78).

Converging evidence has been obtained from within our own rodent stroke study. While no adverse effects (e.g., abnormal changes in body weight) were observed, results indicate that daily exposure to ENTF treatment (7.86 Hz, 1 G) over 8 weeks post-injury significantly improved the Neurological Severity Score (NSS) in the treatment group. Importantly, a significant increase in the number of BrdU positive cells was found in the dentate gyrus, in addition to the restoration of biomarkers indicative of healthy cortical tissue in the injured parietal cortex of ENTF-treated mice. These results further support the hypothesis that ENTF treatment may promote neurogenesis (38). Additionally, in rats with spinal cord injury, diffusion tensor imaging (DTI) revealed that those receiving ENTF treatment showed evidence of structural neuroplasticity, compared to the spinal cord degradation observed in non-treated rats (61).

The substantial number of published studies clearly demonstrate effects from cellular to physiological, and consequently behavioral, suggesting a robust mechanism of action mediating the effect of ELF-EMF on the brain. The present results extend these findings to a clinical post-stroke population, and demonstrate the effectiveness of ENTF treatment in accelerating recovery in the subacute phase post-stroke.

Importantly, the present results provide useful data on the safety and feasibility of ENTF treatment as there were no safety concerns or complaints about comfort. Indeed, a non-invasive, user-friendly device with a favorable safety profile may be ideally suited for use after a patient returns home. Further, in the wake of the COVID-19 pandemic, efficacious treatment options that minimize in-hospital exposure are valuable for an older, vulnerable, post-stroke population. The ability to integrate such treatment into a care plan that is patient-centered and addresses the normally fragmented treatment pathway remains an important target of future studies.

This study has several limitations. First, though results are robust across multiple metrics, sample size was small. In addition, there were limited long-term follow-up evaluations to assess the continued effects of the treatment on recovery. COVID-19 restrictions forced a reduction of the planned sample size and follow-up duration, thus studies with larger samples and longer-term follow-up are needed. Second, the FMA-UE and ARAT measures appeared suboptimal for moderately impaired participants due to reasonable likelihood of reaching the maximum score (79). Indeed, the trend towards meaningful improvement of the FMA-UE (*p* = 0.06) coupled with an overwhelming majority of treated participants in the top 10% of the metric at end of treatment, it is likely that the benefit to the treatment group was not fully captured in this score. In comparison, there was continued improvement on BBT which has a greater responsive range. Third, the study was conducted at a single site in participants of one ethnicity. Multicenter trials in larger, more diverse populations are desirable.

In conclusion, this study demonstrates efficacy of extremely low frequency, low intensity frequency-tuned ENTF exposure in improving upper extremity motor function and reducing disability during the subacute phase in post-ischemic stroke patients. There was clinically meaningful improvement in upper extremity motor function and overall disability reduction as measured by multiple metrics, including FMA-UE, mRS, ARAT, BBT, and NIHSS. Given the high stroke prevalence and limited treatment options beyond the acute phase, these results represent a promising avenue for alternative treatment that non-invasively targets and rehabilitates compromised brain processes, and is applicable to a wide swath of post-stroke patients. The current findings should be extended by examining ENTF treatment in a larger sample with longer follow-up, as well as examining direct indices of plasticity and feasibility of continuing treatment at home. Additionally, future work may explore the generalizability of this approach to other functional domains (e.g., cognitive function), as well as other neurological and neurodegenerative disorders.

## Data availability statement

The raw data supporting the conclusions of this article will be made available by the authors upon reasonable request.

## Ethics statement

The studies involving human participants were reviewed and approved by Dr. B. L. Kapur Memorial Hospital Ethics Committee. The patients/participants provided their written informed consent to participate in this study.

## Author contributions

BW, DP, AL, ES, YS, YD, AA, and NB were all involved in the initial study design and execution plan. DP and AP lead participant screening and recruitment, as well as study execution. AL and OV were responsible for study oversight. OV was additionally responsible for data preparation and validation. BW, JS, AH, AL, ES, YS, SR, and NB contributed to data analysis and presentation. BW, AB, and GD prepared this manuscript. All authors reviewed and approved the final version of the manuscript.

## Funding

This study was sponsored by BrainQ Technologies Ltd.

## Conflict of interest

Authors BW, AH, AB, GD, AL, OV, ES, YS, SR, YD, and AA are/were employed by the study funder, BrainQ Technologies Ltd. Authors ES and YS have ownership interest in BrainQ Technologies Ltd. Authors JS and NB serve as advisors to BrainQ Technologies Ltd. Author YS is the father of a neurologically disabled child. This study received funding from BrainQ Technologies Ltd. The funder was involved in the study design, data analysis and interpretation, and the writing of this article or the decision to submit it for publication. The remaining authors declare that the research was conducted in the absence of any commercial or financial relationships that could be construed as a potential conflict of interest.

## Publisher's note

All claims expressed in this article are solely those of the authors and do not necessarily represent those of their affiliated organizations, or those of the publisher, the editors and the reviewers. Any product that may be evaluated in this article, or claim that may be made by its manufacturer, is not guaranteed or endorsed by the publisher.
