# Supplementary material for: Frequency-tuned electromagnetic field therapy improves post-stroke motor function: A pilot randomized controlled trial
Source: Front Neurol. 2022 Nov 14;13:1004677. doi: 10.3389/fneur.2022.1004677 (PMC9702345; doi:10.3389/fneur.2022.1004677)
Supplement: Supplementary file 1 [file Table_1.DOCX]

**Table S1: Inclusion/Exclusion Criteria**

| **Inclusion Criteria** | **Exclusion Criteria** |
| --- | --- |
| 1. Diagnosis of first ever ischemic stroke confirmed by imaging (or first recurrent stroke with no previous neurological impairment in the same limb before onset of current stroke) 2. 72h to 15 days from stroke onset (or 21 days from stroke onset if patient was still unstable at day 15 from stroke onset) 3. Medically stable 4. Age: >18 and <80 years, independent in daily activities before stroke onset 5. Right hand dominant 6. FM >10 and <45 for affected upper limb. 7. Able to sit in a chair for 70 consecutive minutes and follow three verbal instructions:    1. Raise your healthy arm to the level of your shoulder    2. Pick up this pen with your healthy hand    3. Put your healthy hand on your waist 8. Able to understand the information provided and give informed consent | 1. Contraindication for MRI 2. Recent epileptic seizures or epilepsy and/or currently taking medication known to lower the seizure threshold 3. An acute problem such as an active infection at the time of recruitment 4. Previous disease of the central nervous system 5. Damage to joints, bones and muscles causing restriction of movement of the affected upper limb 6. Suffering from peripheral nerve damage of the affected upper limb 7. Pregnant women and women who may become pregnant unless they are on an acceptable method of contraception throughout the study 8. Alcoholism or drug addiction (as defined by DSM-IV) within previous 5 years |
