# Supplementary material for: Frequency-tuned electromagnetic field therapy improves post-stroke motor function: A pilot randomized controlled trial
Source: Front Neurol. 2022 Nov 14;13:1004677. doi: 10.3389/fneur.2022.1004677 (PMC9702345; doi:10.3389/fneur.2022.1004677)
Supplement: Supplementary file 2 [file Table_2.DOCX]

**Table S2: Participant Demographics, Intention-to-treat (ITT) Set**

|  | **Sham Group (*n*=9)** | **ENTF Group (*n*=15)** | **Total**  **(n = 24)** |
| --- | --- | --- | --- |
| Age, yrs, mean (±SD) | 55.8 (±9.6) | 55.9 (±17.2) | 55.8 (±14.6) |
| Sex, female (%) | 33% | 20% | 25% |
| Race-Ethnicity, South-Asian (%) | 100% | 100% | 100% |
| Hand dominance, right (%) | 100% | 100% | 100% |
| Affected hand, right (%) | 56% | 33% | 42% |
| Time from stroke onset to first treatment,  days, median (IQR) | 15.0  (11.0–19.0) | 10.0  (7.5–13.5) | 12.0  (8.8–15.3) |
| FMA-UE Baseline, mean (±SD) | 19.3 (±8.3) | 27.7 (±11.1) | 24.5 (±10.8) |
| mRS Baseline, mean (±SD) | 3.4 (±0.7) | 3.7 (±0.5) | 3.6 (±0.6) |
