# Supplementary material for: Frequency-tuned electromagnetic field therapy improves post-stroke motor function: A pilot randomized controlled trial
Source: Front Neurol. 2022 Nov 14;13:1004677. doi: 10.3389/fneur.2022.1004677 (PMC9702345; doi:10.3389/fneur.2022.1004677)
Supplement: Supplementary file 3 [file Table_3.DOCX]

**Table S3: Participant Demographics, As Treated (AT) Safety Set**

|  | **Sham Group (*n*=9)** | **ENTF Group (*n*=19)** | **All**  **(n=28)** |
| --- | --- | --- | --- |
| Age, yrs, mean (±SD) | 55.8 (±9.6) | 54.7 (±16.0) | 55 (±14.1) |
| Sex, female (%) | 33% | 21% | 25% |
| Race-Ethnicity, South-Asian (%) | 100% | 100% | 100% |
| Hand dominance, right (%) | 100% | 100% | 100% |
| Affected hand, right (%) | 56% | 42% | 46% |
| Time from stroke onset to first treatment,  days, median (IQR) | 15.0  (11.0–19.0) | 12.0  (8.0–14.0) | 12.5  (9.8–15.3) |
| FMA-UE Baseline, mean (±SD) | 19.3 (±8.3) | 26.1 (±11.2) | 23.9 (±10.7) |
| mRS Baseline, mean (±SD) | 3.4 (±0.7) | 3.8 (±0.6) | 3.7 (±0.7) |
