# Supplementary material for: Frequency-tuned electromagnetic field therapy improves post-stroke motor function: A pilot randomized controlled trial
Source: Front Neurol. 2022 Nov 14;13:1004677. doi: 10.3389/fneur.2022.1004677 (PMC9702345; doi:10.3389/fneur.2022.1004677)
Supplement: Supplementary file 4 [file Table_4.DOCX]

**Table S4. Raw Scores Results Table (Per Protocol Set)***

|  | **Sham Group (*n* = 8)** | **ENTF Group (*n* = 13)** |
| --- | --- | --- |
| ***Primary Outcome Measure*** | | |
| **Fugl Meyer Assessment - Upper Extremity (FMA-UE)** | | |
| Baseline | 18.8±8.7 | 26.8±11.5 |
| Week 4 | 28.4±11.8 | 49.9±14.6 |
| Week 8 | 41.9±19.3 | 58.2±12.9 |
| ***Secondary Outcome Measures*** | | |
| **Modified Rankin Scale (mRS)** | | |
| Baseline | 3.4±0.7 | 3.6±0.5 |
| Week 8 | 2.1±0.8 | 1.2±0.8 |
| **Action Research Arm Test (ARAT)** | | |
| **ARAT Grasp Subscale** | | |
| Baseline | 3.0±6.4 | 7.3±7.3 |
| Week 8 | 11.1±7.6 | 16.4±4.4 |
| **ARAT Grip Subscale** | | |
| Baseline | 1.3±2.8 | 3.7±3.7 |
| Week 8 | 6.6±4.6 | 10.5±3.2 |
| **ARAT Pinch Subscale** | | |
| Baseline | 1.1±2.1 | 1.5±2.3 |
| Week 8 | 6.4±7.0 | 14.8±6.0 |
| **ARAT Gross Movement** | | |
| Baseline | 3.5±1.5 | 5.5±2.0 |
| Week 8 | 7.3±2.0 | 8.4±1.3 |
| **ARAT Total Score** | | |
| Baseline | 8.9±12.1 | 18.0±13.7 |
| Week 8 | 31.4±20.2 | 50.1±14.4 |
| **FMA-Lower Extremity** | | |
| Baseline | 18.9±9.9 | 19.9±7.5 |
| Week 8 | 28.8±8.2 | 33.8±0.8 |
| **Box & Blocks Test (BBT) - Affected Hand** | | |
| Baseline | 4.0±7.5 | 7.5±7.6 |
| Week 2 | 4.5±7.8 | 8.8±8.2 |
| Week 4 | 5.4±8.6 | 14.3±9.8 |
| Week 6 | 7.3±8.3 | 18.5±9.3 |
| Week 8 | 12.5±9.2 | 30.0±12.3 |
| **BBT- Non-Affected Hand** | | |
| Baseline | 28.4±12.4 | 36.6±6.2 |
| Week 2 | 31.3±12.6 | 38.5±5.1 |
| Week 4 | 32.8±12.8 | 45.6±9.4 |
| Week 6 | 36.1±12.6 | 49.0±10.3 |
| Week 8 | 37.8±13.6 | 55.0±12.2 |
| **National Institutes of Health Stroke Scale (NIHSS)** | | |
| Baseline | 10.3±4.3 | 8.7±3.8 |
| Week 8 | 5.5±4.2 | 2.1±2.3 |
| **PROMIS-10, Global Physical Health** | | |
| Baseline | 33.0±2.7 | 35.2±5.1 |
| Week 8 | 41.9±6.3 | 46.2±6.7 |
| **PROMIS-10, Global Mental Health** | | |
| Baseline | 24.7±4.4 | 29.7±7.0 |
| Week 8 | 37.7±10.2 | 44.4±7.3 |

* All values represent raw scores (mean ± SD)
